# Patient satisfaction with eletriptan in the acute treatment of migraine in primary care

**DOI:** 10.1111/j.1742-1241.2007.01513.x

**Published:** 2007-10

**Authors:** R B Nett, P J Tiseo, M Almas, C R Sikes

**Affiliations:** 1 Texas Headache Associates San Antonio, TX, USA; 2 Pfizer Inc. New York, NY, USA

## Abstract

**Summary:**

**Objective:**

The efficxacy of triptans for acute migraine has been well established in clinical trials but not in primary care, where they are most commonly prescribed. The aim of this open-label study was to evaluate the effectiveness of eletriptan 40 mg in primary care, using a patient-weighted satisfaction scale.

**Methods:**

Eligible patients met International Headache Society criteria for migraine, with 1–6 attacks per month. Patients completed questionnaires at screening and following a single eletriptan-treated attack. Treatment satisfaction was evaluated using a six-item Medication Satisfaction Questionnaire (MSQ). MSQ item scores were weighted, based on the important score ratings, to yield individualised satisfaction scores. The primary end-point was the difference in weighted satisfaction scores between the patient's previous treatment and eletriptan 40 mg. Secondary end-points assessed quality of life (QOL), functioning and efficacy of treatment.

**Results:**

Of 590 patients screened, 437 completed the study. Degree (95.2%), time (88.8%) and duration (83.8%) of headache pain relief were rated as most important by patients. The mean (±SD) total satisfaction score on the MSQ was higher for eletriptan than previous therapy (2.2 ± 3.0 vs. 0.6 ± 2.4; p < 0.001). The high level of satisfaction with eletriptan vs. previous treatment reflects the improvements in QOL and functioning observed, and the high headache and pain-free response rates.

**Conclusions:**

Patient-weighted satisfaction with eletriptan 40 mg was higher than with previous treatment for all items. The use of patient-weighted importance ratings of satisfaction is a promising approach for establishing effectiveness of treatment in primary care.

**What's known**

The efficacy of triptans (5-HT_1B/1D_-receptor agonists) has been well established in clinical trials, but not in a primary care setting, where they are most commonly prescribed. Previous studies have highlighted the treatment outcomes that are most important to migraine sufferers (e.g. degree of pain relief, speed of relief and duration of relief). Asking patients to rate their treatment in terms of the treatment outcomes that are most important to them has been suggested as a useful way of assessing the effectiveness of a treatment in primary care.

**What's new**

To our knowledge, this is the first trial to prospectively assess patient-rated satisfaction with treatment as an *a priori* primary outcome. In addition to assessing the utility of such a methodology, this article also discusses the effectiveness of eletriptan for treating acute migraine in the primary care setting.

## Introduction

The efficacy of the triptans (5-HT_1B/1D_-receptor agonists) in treating acute migraine attacks has been established from the results of approximately 100 double-blind, randomised, parallel-group trials (1–3). The current challenge facing physicians is how to optimise the benefits of proven migraine treatments for individual patients.

Placebo-controlled efficacy trials conducted in academic and specialty centres do not always translate into primary care. Furthermore, typical migraine efficacy trials do not address a wide range of common issues such as treatment non-compliance, changing physician, drug switching and the tendency for many patients to discontinue medical management completely (4–7). For these reasons, there has been an increasing interest to move beyond traditional efficacy outcomes to focus on patient-centred measures, such as quality of life (QOL) and functioning.

A patient-centred approach to measuring efficacy in migraine treatment studies involves asking each patient to rate the relative importance of key outcomes, such as speed of pain relief, duration of relief (i.e. absence of headache recurrence), improvement in migraine-associated symptoms (e.g. nausea, photophobia and phonophobia), and risk of side effects. This method for rating the importance to the patient of each clinical outcome has been proposed (8–10), but to our knowledge, has not been used prospectively in a migraine trial.

The aim of this open-label study was to investigate the utility of patient-weighted outcomes for evaluating the effectiveness of eletriptan in the acute treatment of migraine, in a primary care setting.

## Patients and methods

### Patients

Men and women aged 18–65 years were eligible for inclusion in the study if they met International Headache Society criteria for migraine with or without aura (11), with an attack frequency of 1–6 migraines per month, an onset of migraine prior to age 50, and a minimum illness duration of 1 year. Women were required to be postmenopausal, surgically sterile or using a medically accepted form of contraception.

Key exclusion criteria were: (i) the presence of migraine with prolonged aura, familial hemiplegic migraine or migrainous infarction; (ii) any acute or unstable medical condition, clinically significant laboratory test or electrocardiography (ECG) abnormality, or presence of any illness or treatment known to be a contraindication to the safe use of eletriptan as summarised in the US prescribing information label; (iii) the misuse or abuse of alcohol or other substances, based on the Diagnostic and Statistical Manual of Mental Disorders Version IV (DSM-IV) criteria; and misuse of analgesics (defined as use of > 50 g of aspirin or > 100 tablets of analgesics) or ergotamine (defined as use on > 2 days per week).

### Study design

This open-label, single-attack, outpatient study was conducted at 185 primary care practices in the USA between August 2003 and May 2004.

At the screening visit, patients with a history of disabling headaches completed the three-item ID Migraine™ (Pfizer, Inc, New York, NY, USA) questionnaire, a validated self-administered instrument that assesses the presence of nausea, photophobia and headache-related disability (12). Those who scored positively on at least two of the three items were familiarised with the study and asked to provide written informed consent.

To assess their perceptions of previous treatments used, patients were asked to complete three baseline questionnaires at the screening visit: (i) Migraine Relief Questionnaire (MRQ), (ii) Medication Satisfaction Questionnaire (MSQ) and (iii) Migraine Quality of Life Questionnaire (MQOLQ). The MRQ and MSQ are shown in Table 1. The MRQ assessed the relative importance of six treatment outcomes to the patient. The MSQ assessed patient satisfaction with their usual migraine therapy on these six outcomes, and the results of the MRQ were used to weigh the outcomes per patient. The MSQ is based on previous research that identified efficacy-related determinants of patient satisfaction (5,13). The MQOLQ is a validated instrument that asks patients to retrospectively answer 15 questions relating to QOL associated with the 24-h period following treatment of their last migraine (14,15).

**Table 1:** The Migraine Relief Questionnaire and the Medication Satisfaction Questionnaire

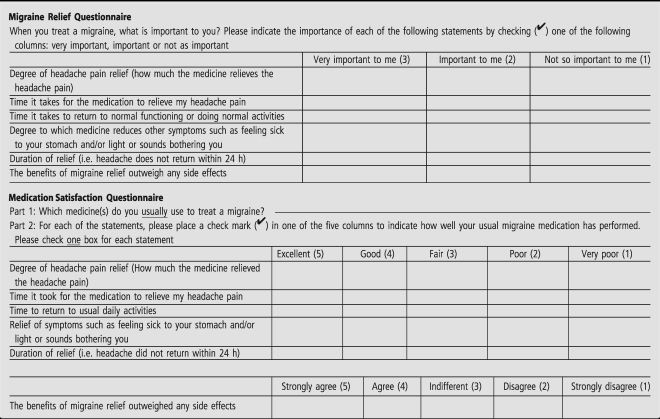

A medical evaluation consisting of a physical examination, measurement of vital signs, a 12-lead ECG and urine pregnancy testing (as appropriate) was also conducted at screening.

Enrolled patients were provided with two tablets of eletriptan 40 mg and a headache diary. Patients were instructed to treat a single migraine attack in the following 12 weeks with eletriptan 40 mg, taking it as soon as they were certain they were experiencing a migraine, after the aura phase (if present) had ended and the headache phase had begun (within 2 h of onset, if possible). If migraine symptoms recurred within 24 h postdose, a second dose of eletriptan 40 mg was allowed, provided at least 2 h had elapsed after taking the first dose. Rescue medication was also permitted if subjective headache relief was inadequate.

Patients recorded the date and time of onset of their eletriptan-treated migraine immediately postattack, and completed two questionnaires: (i) the MQOLQ, to assess QOL associated with eletriptan use 24 h after first dose and (ii) the Functional Assessment in Migraine, Activities and Participation Scale (FAIM-A&P), a validated scale derived from the World Health Organization's International Classification of Impairments, Disability and Handicaps (ICIDU-2) (16), which assesses the degree to which, on a seven-point scale (where seven = impaired none of the time and one = impaired all of the time), migraine-associated impairment affects patient functioning whilst carrying out common activities. The FAIM-A&P was completed at baseline, 2 and 4 h postdose. Patients also recorded headache pain severity, as rated on a four-point scale (where 3 = severe and 0 = no pain), at baseline, 2 and 24 h postdose, and noted whether a second dose of eletriptan or any rescue medication was taken.

Patients were instructed to return to the study centre within 2 weeks of their eletriptan-treated attack to complete two further questionnaires: (i) the MSQ, to assess treatment satisfaction with eletriptan 40 mg and (ii) a migraine treatment preference questionnaire, which asked patients about their overall migraine medication preferences.

At study conclusion investigators rated, on a seven-point scale (where one = very dissatisfied and seven = very satisfied), their satisfaction with ID Migraine™ as a tool to: (i) identify migraine sufferers, (ii) help patients to clearly and effectively communicate their symptoms and (iii) improve patient–physician dialogue related to headache diagnosis.

### Study assessments

The primary end-point was the difference in satisfaction between a patient's previous migraine treatment and eletriptan 40 mg, as measured on the six weighted items of the MSQ. The secondary efficacy end-points were: (i) the MQOLQ; (ii) the FAIM-A&P; (iii) 2-h headache response, defined as improvement in headache intensity to mild or no pain from a pretreatment level of moderate or severe, rated on a four-point global intensity scale (no pain, mild, moderate and severe); (iv) 2-h pain-free response, defined as improvement to no pain at 2 h postdose following a pretreatment pain level of moderate or severe, on the four-point scale; (v) sustained headache response at 48 h postdose, defined as response within 2 h of the first dose of study medication, no headache recurrence, no use of rescue medication and no second dose of eletriptan within the remainder of a 48-h period; (vi) sustained pain-free response at 48 h defined as pain-free response within 2 h of the first dose of study medication, no headache recurrence, no rescue medication use and no second dose of eletriptan within the remainder of a 48-h period; (vii) patient drug preference, as indicated by the migraine treatment preference questionnaire; and (viii) investigator satisfaction with the ID Migraine™ screener. Tolerability was also assessed.

### Statistical analyses

As this was an open-label, single-attack study, descriptive statistics were calculated, but no significance testing was performed.

The averaged MSQ scores were weighted based on the importance values assigned to specific treatment attributes at the screening visit using the three-point MRQ. The MSQ item scores were multiplied by one if the patient rated the outcome item as ‘not so important’, two if the patient rated the item as ‘important’ or three if the patient rated the item as ‘very important’. To aid in the interpretation of the satisfaction score, the MSQ scores were re-coded from a one to five rating to a −6 to +6 rating, where the lowest number corresponded to a rating of ‘very poor’ and the highest number corresponded to a rating of ‘excellent’. Descriptive statistics (mean and standard deviation [SD]) for the weighted scores and the weighted difference scores were calculated.

Descriptive statistics were performed on the MQOLQ individual and the total domain scores, and a paired *t*-test was used to assess the change from baseline to 24 h postdose.

A standard score was calculated for each patient from the FAIM-A&P scale as follows: (i) the rating scale was reversed (one = impaired none of the time; seven = impaired all of the time) and (ii) the score on each item was summed to obtain a raw total score. The raw total score was subsequently transformed to a standardised score of 0–100, with a higher value indicating improved functioning. A paired *t*-test was performed to test the change from baseline to 2 and 4 h postdose.

The study was conducted according to the Declaration of Helsinki (1996 revision) and is consistent with the International Conference on Harmonization Good Clinical Practice guidelines (17). The protocol was approved by Ethic Committees at each site.

## Results

Five hundred and ninety patients were screened, of whom 582 met eligibility criteria and were entered into the study. The safety sample consisted of 481 patients who took study medication, while the intent-to-treat sample consisted of all patients who took study medication and completed the MSQ postdose (*n* = 437).

The baseline characteristics of the safety sample are summarised in Table 2. The preponderance of women (85%), and individuals in the 30- to 50-year-old age range, is typical of most migraine clinical trials. Patients in the study reported an extensive array of previous migraine therapies, and many used a combination of therapies. Most patients utilised non-migraine specific acute therapies such as non-steroidal anti-inflammatory drugs (NSAIDs) (31%) and non-NSAID analgesics (61%). Migraine specific drugs (triptans and ergotamine-containing preparations) were used by 47% of study patients.

**Table 2:** Demographic and clinical characteristics of the patient population (*n* = 481)

**Patient characteristics**	
Female, %	85
**Age, years**	
Mean ± SD	39.1 ± 10.6
Range	17—65
**Race, %**	
White	79
Black	11
Others	10
**Aura subtype, %**	
Without aura	47
With aura	29
Mixed	24
Attack frequency in past 3 months, mean ± SD	8.1 ± 4.8
Previous acute migraine therapy*	
NSAIDs, %	31
Non-NSAID analgesics, %	61
Migraine-specific treatment (triptans, ergot-containing drugs), %	47

*Patients may have used more than one class of therapy. NSAID, non-steroid anti-inflammatory drug.

The MRQ, completed at the screening visit, provided data on the relative importance of key clinical outcomes to the patients. The degree of pain relief was viewed as very important by the highest proportion of patients (95.2%), followed by time of pain relief (88.8%) and duration of pain relief (83.8%) (Table 3). Results for each patient were used to provide individual weighting to the MRQ satisfaction items.

**Table 3:** Relative importance of clinical outcomes: results from Migraine Relief Questionnaire at screening (*n* = 437)

	Relative importance		
Items	Very important	Important	Not so important
Degree of headache pain relief, *n* (%)	416 (95.2)	18 (4.1)	3 (0.7)
Time of pain relief, *n* (%)	388 (88.8)	47 (10.8)	2 (0.5)
Duration of relief, *n* (%)	366 (83.8)	68 (15.6)	3 (0.7)
Time to return to usual activities, *n* (%)	349 (79.9)	85 (19.5)	3 (0.7)
Relief of migraine-associated symptoms, *n* (%)	317 (72.5)	111 (25.4)	9 (2.1)
Efficacy of migraine treatment outweighs side effects, *n* (%)	263 (60.2)	153 (35.0)	21 (4.8)

### Patient satisfaction on the MSQ

Treatment with eletriptan 40 mg vs. usual previous treatment was associated with higher weighted satisfaction scores overall and on the six individual items of the primary outcome measure, the MSQ (Table 4).

**Table 4:** Mean weighted Medication Satisfaction Questionnaire satisfaction scores for six items: eletriptan 40 mg vs. immediate previous migraine treatment

Items	Immediate previous treatment score	Eletriptan 40 mg score	Difference in score	p-value
**Overall satisfaction scores**	0.6 ± 2.4	2.2 ± 3.0	1.6 ± 3.9	< 0.001
Degree of headache pain relief, *n* (%)	1.2 ± 3.1	2.6 ± 3.5	1.4 ± 4.7	< 0.001
Time of pain relief, *n* (%)	0.2 ± 3.0	1.8 ± 3.5	1.6 ± 4.6	< 0.001
Duration of pain relief, *n* (%)	−0.1 ± 3.2	2.2 ± 3.9	2.3 ± 4.9	< 0.001
Time to return to usual activities, *n* (%)	−0.1 ± 2.8	1.8 ± 3.1	1.9 ± 4.4	< 0.001
Relief of migraine-associated symptoms, *n* (%)	0.0 ± 3.0	2.2 ± 3.3	2.2 ± 4.5	< 0.001
Efficacy of migraine treatment outweighs side effects, *n* (%)	2.5 ± 2.7	2.8 ± 3.1	0.3 ± 3.6	0.0866

**Figure 1:**
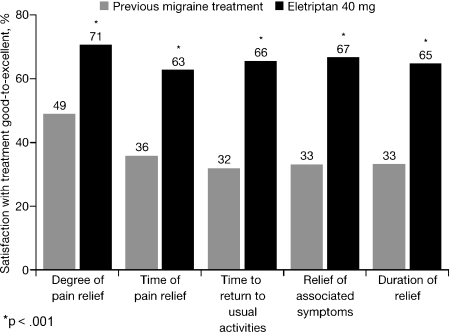
Proportion of patients reporting satisfaction as ‘good-to-excellent’: comparison of eletriptan 40 mg with previous migraine treatment (total sample, *n* = 437)

For the total sample, the proportion of patients reporting treatment satisfaction as ‘good-to-excellent’ was significantly higher on eletriptan compared with usual previous treatment on the first five MSQ treatment satisfaction items (p < 0.001; Figure 1). The proportion of patients reporting ‘agree-to-strongly agree’ that the benefits of treatment outweighed the side effects was also higher on eletriptan than previous treatment, although this was not significant (79% vs. 72%).

**Figure 2:**
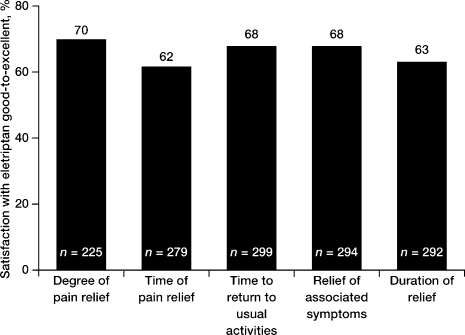
Subgroup analysis: proportion of patients reporting ‘fair-to-very poor’ response to previous migraine treatment who reported satisfaction as ‘good-to-excellent’ when switched to eletriptan 40 mg

**Figure 3:**
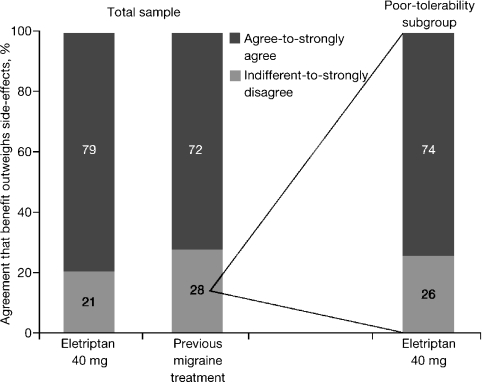
Do the efficacy benefits of therapy outweigh the side effects? Results for total sample and the poor tolerability subgroup that switched to eletriptan 40 mg from previous migraine treatment

Approximately 50% of patients reported ‘fair-to-very-poor’ satisfaction with previous migraine therapy across each of the MSQ items. Treatment with eletriptan resulted in a high level of ‘good-to-excellent’ satisfaction on each of the MSQ items in this subgroup (62–70%; Figure 2). Similarly, in the subgroup of patients (28%) who reported that the efficacy benefits of their previous migraine therapy did not outweigh the side effects, 74% of them changed their rating to ‘agree-to-strongly agree’ after switching to eletriptan treatment (Figure 3).

### Effect of eletriptan on quality of life and functioning

Treatment with eletriptan was associated with greater improvement in all aspects of QOL, compared with previous migraine therapies, as measured using the MQOLQ (Table 5).

**Table 5:** Mean (±SD) improvement in Migraine Quality of Life Questionnaire (MQOLQ) domain scores: comparison of the effect of previous and current treatment on quality of life (*n* = 416)

MQOLQ domain	Previous treatment	Eletriptan 40 mg
Symptoms*	+10.3 ± 4.5	+15.2 ± 4.9
Feelings/concerns	+8.7 ± 4.5	+13.8 ± 5.6
Work*	+10.2 ± 4.8	+14.4 ± 5.7
Social/interpersonal	+9.7 ± 4.8	+14.1 ± 5.5
Energy/vitality	+9.0 ± 5.0	+13.7 ± 5.9

*Sample size was smaller for the symptoms domain (*n* = 415) and the work domain (*n* = 414).

At the time of taking eletriptan, the mean (±SD) FAIM-A&P score was 23.2 ± 23.3, a score consistent with clinically significant migraine-related impairment in functioning. At 2 h postdose, the mean FAIM-A&P score had increased by 31.5 points to 54.7 ± 34.5, thus functional impairment was significantly reduced (p ≤ 0.001). At 4 h, the score showed a further increase to 67.7 ± 36.4.

### Efficacy evaluation

For the total sample (*n* = 437), 62% [95% confidence interval (CI): 51–60%] of patients experienced 2-h headache response and 36% (95% CI: 31–40.3%) experienced 2-h pain-free response. Headache response was sustained over 48 h in 33% (95% CI: 25–46%) of patients, and a pain-free response was sustained in 23% (95% CI: 14–31%).

### Patient preference

Of 426 patients who provided preference data, 254 (59.6%) preferred eletriptan 40 mg to all previous acute migraine treatments they had used.

### Investigator satisfaction with ID Migraine™

One hundred and forty-three investigators rated their satisfaction with the ID Migraine™ screener at study conclusion. Satisfaction was extremely high on all items assessed. One hundred and twenty-nine investigators (90%) were ‘satisfied’ or ‘very satisfied’ with the assistance provided by ID Migraine™ in identifying migraine sufferers, 126 (88%) were ‘satisfied’ or ‘very satisfied’ with how ID Migraine™ helped their patients to clearly and effectively communicate their symptoms, and 129 (90%) were ‘satisfied’ or ‘very satisfied’ that ID Migraine™ improved patient–physician dialogue related to headache diagnosis.

### Tolerability and safety

Only one adverse event, nausea (2.1%), occurred with an incidence ≥ 2%. Overall, 10.8% of patients on eletriptan reported having at least one adverse event. Five patients (1.0%) rated their adverse events as ‘severe’. There was one serious adverse event, which was not treatment related (a patient was involved in a motor vehicle accident 3 days after taking eletriptan). No patients discontinued treatment because of an adverse event. There were no clinically significant changes in laboratory tests, vital signs or ECG during the study.

## Discussion

We report here the results of an open-label, single-attack study that used patient-centred ratings of treatment satisfaction as the primary outcome. To our knowledge, no previous trial has utilised weighted satisfaction to assess the primary, *a priori* end-point.

The MRQ indicates that the three most important treatment outcomes for patients were degree of pain relief (95%), time of pain relief (89%) and duration of relief (84%). These results reflect those reported by Lipton and Stewart (5) with complete pain relief (87%), no recurrence (86%) and rapid onset of relief (83%) rated most highly by migraine sufferers. A study of 438 physicians asked to evaluate 12 attributes of acute migraine treatment also rated degree of pain relief (22%) and rapid onset of relief (15%) most highly (18). Therefore, both patients and physicians agree that it is most important that an effective migraine treatment eradicates pain completely, reduces pain rapidly and has a long-lasting effect.

Our study found treatment with eletriptan 40 mg was associated with high levels of satisfaction in approximately two-thirds of patients for degree of pain relief, time of pain relief, duration of relief, time to return to usual activities and relief of associated symptoms. In contrast, only 49% of patients reported high levels of satisfaction with previous treatment for degree of pain relief, and only one-third of patients reported high levels of satisfaction for the other outcomes.

The high satisfaction reported with eletriptan 40 mg most likely reflects the high 2-h and sustained (48-h) headache response, and pain-free response rates recorded. We would expect presence/absence of a 2-h headache/pain-free response to influence satisfaction with time of pain relief and time to return to usual activities, presence/absence of pain-free response (at 2 and 48 h) to influence satisfaction with degree of headache pain relief, and presence/absence of sustained headache/pain-free response to influence satisfaction with duration of relief. Indeed, the 2-h headache response rate (62%) and the proportion of patients reporting satisfaction as ‘good-to-excellent’ for time to headache pain relief (63%) are approximately equal. The high levels of satisfaction reported by patients treated with eletriptan also reflect improvements in both QOL, as measured by the MQOLQ, and in functioning, as measured by the FAIM-A&P scale. In particular, improved QOL and functioning would impact on satisfaction with time to return to usual activities, which was rated as ‘good-to-excellent’ in 66% of study participants.

Eletriptan was also effective in achieving high levels of satisfaction in patients reporting low satisfaction with previous migraine therapy and in patients who experienced poor tolerability. Thus, switching a patient from a suboptimally effective triptan to eletriptan can be a useful treatment strategy, a finding consistent with those of previous switch studies (19,20).

The study was limited by the following important factors: (i) the study was not double blind, and had no parallel-group active comparator or placebo control; rather it was an open-label study specifically focusing on satisfaction with eletriptan 40 mg. Open-label studies may be affected by patients’ views about the study medication, whether positive or negative. The fact that eletriptan is a new treatment may also affect the way it is perceived relative to longer established therapies, (ii) patient perceptions of eletriptan treatment were based on only one treated attack, which may or may not have been ‘typical’ for that patient, (iii) patient ratings of their previous migraine treatment were retrospective, which may have introduced recall bias, (iv) furthermore, although patients were asked to rate their ‘usual’ therapy, their response may have been influenced by their experience of a number of treatments, not just their most commonly utilised therapy. Thus the results of the baseline MSQ may reflect general impressions with previous treatments used and (v) we did not evaluate whether patients on different previous treatments reported different satisfaction results with eletriptan. A patient's prior experience with treatment may affect their rating of a new therapy. For example, patients previously receiving migraine-specific drugs such as triptans or ergotamine may have had higher expectations of eletriptan than patients previously treated with general analgesics.

Despite these limitations, our study demonstrates the utility of patient-weighted satisfaction scores in assessing migraine treatment effectiveness. It also shows that eletriptan 40 mg produces higher levels of satisfaction than previous migraine therapy on the items identified as important to patients. The preference weighting methodology used in the current study is a promising approach for measuring patient-rated outcomes, as it customises standard efficacy assessments based on individualised patient inputs. Consequently, the real-world effectiveness of treatment can be assessed on an individual patient basis in terms of the factors that are most important to them.
